# Toward a translational team science hierarchy of needs: Exploring the information management challenges of team science

**DOI:** 10.1017/cts.2023.614

**Published:** 2023-08-23

**Authors:** Patrick W. Kelly, Jason Chladek, Betsy Rolland

**Affiliations:** 1 Institute for Clinical and Translational Research, School of Medicine and Public Health, University of Wisconsin-Madison, Madison, WI, USA; 2 Social and Administrative Sciences Division, School of Pharmacy, University of Wisconsin-Madison, Madison, WI, USA; 3 Carbone Cancer Center, School of Medicine and Public Health, University of Wisconsin-Madison, Madison, WI, USA

**Keywords:** Information behaviors, information management, translational teams, clinical and translational research, team science

## Abstract

**Background:**

Clinical and Translational Research (CTR) requires a team-based approach, with successful teams engaging in skilled management and use of information. Yet we know little about the ways that Translational Teams (TTs) engage with information across the lifecycle of CTR projects. This qualitative study explored the challenges that information management imposes on the conduct of team-based CTR.

**Methods:**

We conducted interviews with ten members of TTs at University of Wisconsin. Interviews were transcribed and thematic analysis was conducted.

**Results:**

TTs’ piecemeal and reactive approaches to information management created conflict within the team and slowed scientific progress. The lack of cohesive information management strategies made it more difficult for teams to develop strong team processes like communication, scientific coordination, and project management. While TTs’ research was hindered by the institutional challenges of interdisciplinary team information sharing, TTs who had developed shared approaches to information management that foregrounded transparency, accountability, and trust, described substantial benefits to their teamwork.

**Conclusion:**

We propose a new model for the Science of Team Science field – a Translational Team Science Hierarchy of Needs – that suggests interventions should be targeted at the appropriate stage of team development in order to maximize a team’s scientific potential.

## Introduction

The most pressing societal problems of the 21^st^ century will increasingly require an innovative team science approach [1,2]. While the conduct of high-impact, interdisciplinary science has many benefits, including the integration of knowledge from diverse fields and higher publication and citation rates, it is not without its challenges [3]. The Science of Team Science (SciTS) field has shown how effective science teams benefit from a number of team processes, such as identifying clear roles and responsibilities for its members as well as crafting collaboration plans and authorship agreements [4]. Further, the emerging field of Translational Science, defined as the study of the “translational process in order to establish its governing scientific principles [5],” has identified best practices and approaches to the conduct of Translational Research [6,75]. We argue that what unites these evidence-based practices is a team’s skilled management and use of *information*, which we define here as the human-generated digital objects that teams use to create new biomedical knowledge [8].

However, little attention has been paid by either the SciTS or Translational Science fields to how members of translational teams (TTs) share and manage their information. This lack of attention has an outsized impact on the umbrella enterprise of Clinical and Translational Research (CTR) which is, by definition, a team-based endeavor, one that brings together the perspectives of basic scientists, clinicians, population scientists, community partners, and other stakeholders [9]. TTs fuse together academic research and product development with the goal of advancing a discovery from the bench to the clinic to the community [6]. Hailing from a diverse array of disciplines, institutions, and professions, TT members have been usefully synthesized as twelve *personas*, or roles, by Gonzales *et al*. (2020), including (but not limited to): Basic Scientist, Biostatistician, Clinical Research Center Administrator, Clinical Research Coordinator, Community-Engaged Researcher, Data Analyzer, Developer, K Scholar, Librarian, Patient Navigator, Physician Scientist, and Research Administrator [10]. While this diversity of personas allows for scientific synergies, it also may give rise to the challenge of integrating approaches to information management across disciplinary, professional, and personal boundaries [7,11].

As we consider how each of the twelve personas of TTs works with a wide variety of types of information, the serious challenges of promoting sound information management practices begin to emerge. For example, each piece of information is generated, stored, retrieved, and discussed in a variety of different ways by different team members, both individually and collectively, and at different points in the project. At the same time, information is critical to the conduct of research, protection of human subjects, and accurate reporting to regulators and the scientific community. The transient nature of TT membership presents an additional challenge, as new members must be onboarded and oriented to a team’s information management practices without the loss of historical knowledge from departing members. Previous work by our team has shown that teams too often rely on faulty collective memory when information is missing, which can produce mistakes in a team’s documentation and cause unwanted delays in team progress [12]. Further, data sharing and reuse are impossible when data sets are compromised by poor study or data documentation [13].

Each individual on a TT brings a personal approach to information management, often developed over time and applied successfully to their own work. While these approaches may work for an individual, they are rarely designed to scale and generally conflict with the personal approaches of other teammates. Research has shown that knowledge workers spend approximately 19% of their time finding information needed to complete their tasks [14]. On TTs, our previous work has revealed that they use a constellation of tools and approaches simultaneously, and, often, haphazardly, including file-sharing software (e.g., Box, Google docs), electronic lab notebooks, shared network drives, texting, email, phone calls, and in-person conversations [15]. Multiplied across a large team of researchers, this wasted time represents a substantial burden for busy teams and a regrettable loss of scientific progress.

To our knowledge, this is the first study to delve into the ways that TTs engage with information across the lifecycle of CTR projects. We draw on a sizable literature in the field of Library and Information Science that examines the *information behaviors* of different professions as they seek, use, create, share, store, and retrieve information [16]. In order for teams to maximize their performance, they need to develop a unified approach to *information management*. As such, we see information management as a *sociotechnical* problem in that it involves both the information behaviors of team members and the technologies, broadly construed, that they use to support those behaviors [17].

Information sharing directly improves a number of team performance factors, including cohesion, member satisfaction, and knowledge integration [18]. A meta-analysis of information sharing on some 3,800 teams in 71 empirical studies tells us they benefit from information-sharing practices in two ways: (1) by expanding the *uniqueness* of available information by pooling its members’ knowledge, and (2) by ensuring the *openness* of information so that members can exchange it as easily as possible. But we know very little about how to promote information sharing on *science teams,* in particular, a knowledge gap our study begins to address. It is especially critical to fill this gap in CTR, because how a team shares information will affect its ability to build strong team processes, which include a team’s overall approach to communication, scientific coordination, and project management [6].

Previous work by our team has shown that information management on TTs is an acute problem because every member acts as a *freelance information management agent*: they make individualized decisions about what tools to use and how to use them, often in a piecemeal and reactive manner [19]. Here, we outline the specific implications these information management challenges have on the conduct of team science and on the design of SciTS interventions to mitigate them.

Four main themes emerged from our data, which we explore here:

1. TTs’ piecemeal, reactive approach to information management created conflict within the team and slowed scientific progress.
2. TTs’ lack of cohesive information management strategies made the development of strong team processes like communication, scientific coordination, and project management more difficult.
3. TTs’ research was hindered by the institutional challenges of interdisciplinary team information sharing.
4. TTs benefitted from shared approaches to information management that foregrounded transparency, accountability, and trust.

Combined, these themes paint a picture of TTs struggling to create solutions to information management challenges in a way that both wastes time and effort but also creates conflict among team members, slowing scientific progress.

## Materials and Methods

We conducted qualitative interviews with members of Clinical and Translational Research (CTR) teams employed by the University of Wisconsin-Madison (UW-Madison). The UW-Madison Office of the Vice Chancellor for Research and Graduate Education – whose mission includes supporting multidisciplinary research centers and institutes – oversees $1.3 billion in annual research expenditures, putting UW-Madison among the top 10 in the nation among universities for volume of research. The Institute for Clinical and Translational Research (UW-ICTR) serves as the UW-Madison Clinical and Translational Sciences Award (CTSA), facilitating CTR across the university. The protocol for this study was deemed exempt by the University of Wisconsin IRB.

We identified members of TTs using research networks within the UW School of Medicine and Public Health, literature searches, the UW-Madison researcher database, and UW-ICTR’s databases of past training and funding awardees. Potential participants received an email (Appendix A) from the research team describing the study and participation requirements. Participants were emailed in four rounds of 10, with 42 invited participants reached in total (two email addresses were no longer in service). Each round of 10 included a mixture of invitations to Principal Investigators and/or Academic Faculty, Research Specialists or Scientists, Program Managers, and Postdoctoral Researchers or Graduate Students. The goal of this recruitment method was to include participants that represented as many of the 12 CTR *personas* identified by Gonzales *et. al* (2020) as possible [10]. We received 11 responses and scheduled 11 interviews; however, one individual canceled their interview and did not respond to a request to reschedule, resulting in 10 interviews.

**Figure 1.**
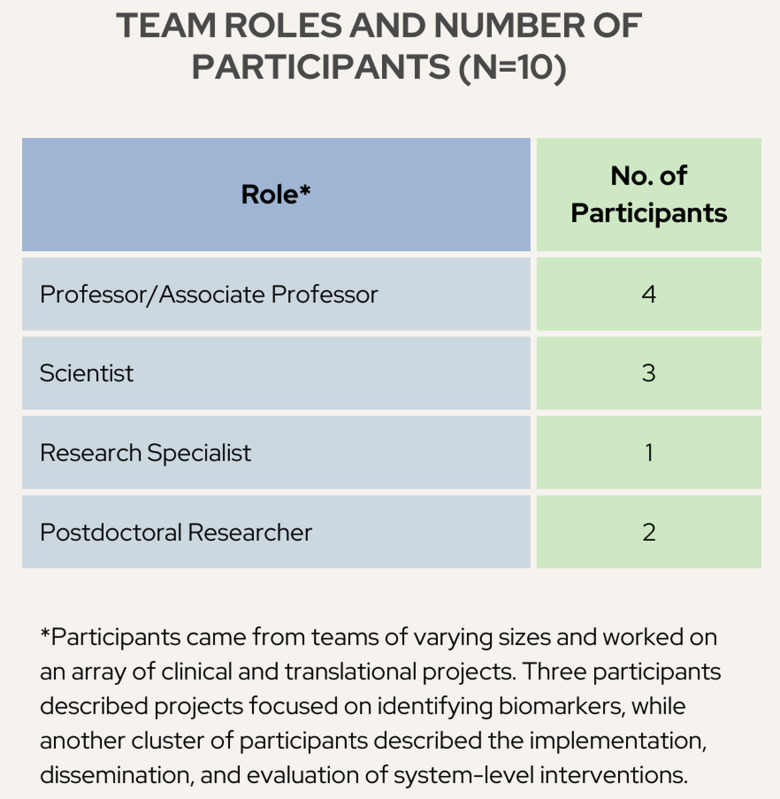
Number of participants by role on translational research team.

Ten participants were included in the final study, as outlined in Fig. 1. Participants hailed from the School of Pharmacy, Department of Family Medicine and Community Health, Ophthalmology, Nutritional Science, Dermatology, Neurology, and Industrial and Systems Engineering. All participants were working on at least one grant-funded CTR project. Interviews included semi-structured questions (Appendix B) related to the overall goal of their research projects and the activities and tools required to complete specific research tasks. Each participant was interviewed once for one hour by a member of the study team, either J.C. or B.R. All interviews took place via Zoom and were video and audio recorded.

The interviews were transcribed verbatim, and the participants were given pseudonyms. Two coders (J.C. and P.K) performed coding of interview transcripts and were supervised by the principal investigator (B.R.), who has experience in qualitative approaches. Coding was conducted via NVivo 12 software (released in 2018) [20]. The transcripts were coded across three rounds. First, the coders performed structural coding based on the interview questions. Second, transcripts were coded for barriers and facilitators under five domains constructed by the researchers (J.C. and P.K.): mental models; team structure, culture, and environment; project management; external factors; and tools. Third, transcripts were coded by research activity (e.g., exploring and defining research topics; building research support systems; holding team meetings; sharing results; and transferring knowledge); these domains were constructed based on our review of the literature on the information behaviors of teams and a modified version of the “information seeking behaviour model” developed by Salajegheh & Hayati (2009) [21]. Coders discussed and resolved coding discrepancies during weekly meetings. Finally, coders reviewed coded data to develop emergent themes, which were discussed with the research team. The coders selected representative quotes for each theme.

## Results

**Figure 2.**
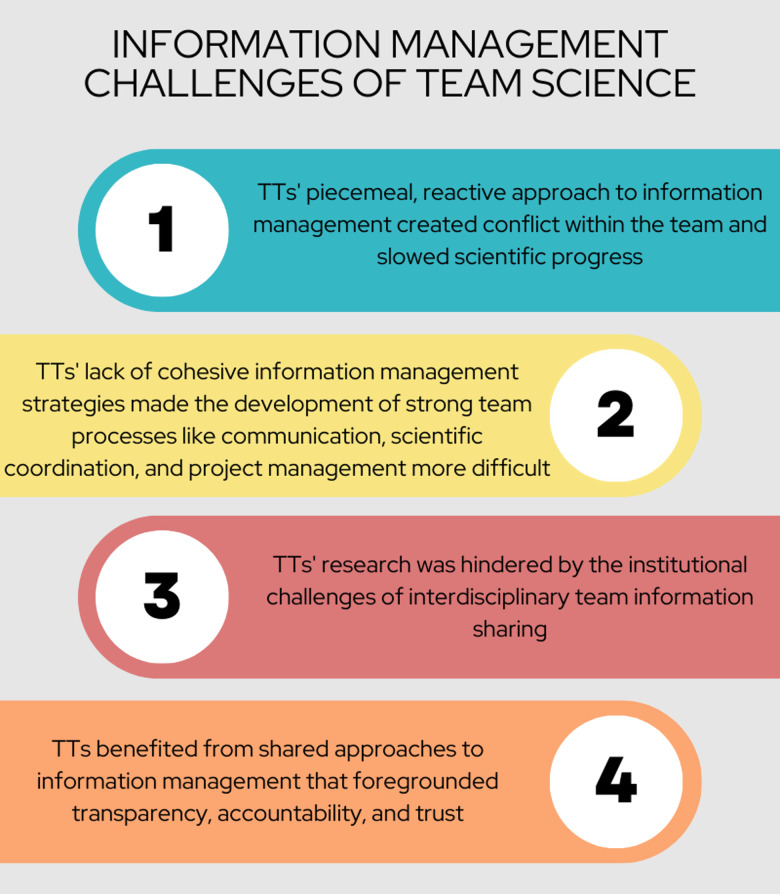
Information management challenges of team science. TT = Translational Teams.

Four themes emerged from our qualitative interviews, as summarized in Fig. 2 and depicted in Table 1. Collectively, they describe how the individual choices of TT members, made in a vacuum without guidance or support from the university, clashed at the team level. These conflicts made the development of strong team processes like communication, scientific coordination, and project management more difficult, invariably slowing scientific progress on their teams.

**Table 1.** Representative participant interview quotes organized by theme. TT = Translational Teams.

Theme	Representative participant interview quote
1. TTs’ piecemeal, reactive approach to information management created conflict within the team and slowed scientific progress	Ben: • “I’ll say that each one of my staff members seems to have a preference for one thing over another. Which, again, is problematic. I have one individual who really has everything nicely organized on the things that they’ve overseeing in Box. I have another individual that keeps everything on the radiology server. And then I have another individual who, uh, or 2 individuals who do kind of a hybrid type system, you know.” (508–512) • “These are the things, these things that we’re talking about, are the things that keep me up at night that no one ever talks about when you’re talking about conducting research. It’s just like all these awesome results, and then you get the publication. But, there’s all these really tedious and challenging steps, right, you know, and I’m not good at too many of them.” (514–518) • “I’ve talked about, to my team recently, about how I’m doing, and I feel like I’m doing less and less science and more of just this type of, you know, administration, budgeting, that kind of thing. I think the longer that I’ve been here, the less and less science I’m actually doing.” (532–534) Alex: • “I like the idea of, like, if I am working with a group on projects to be able to…everything is reachable. From starting with literature to, you know, results, and even the writing process can be evolved there slowly. I can put one piece. You can put once piece. Those are good ideas. But so far, there’s not really any platform successful to do that, or we don't know because it’s, it’s constantly emerging.” (242–246) Katie: • “Interesting question because I don't think we really talked about how we’re going to share it within the team. It just naturally happened. Someone just started using PPT slides, so that’s when we started using PPT slides. And someone tried to share it through Google Drive or Box, and then we started using Box as a platform to share it.” (135–138) • “We didn't really explicitly care about transparency, but then we realized transparency really matters. So, we use a Google Excel sheet to make sure, like, everyone knows what I did and what others did so that we can collaborate with each other…like what we’re going to do with the stored data. Yeah, data entry and verification stuff, and also making sure everything’s really uploaded to the right place and named correctly.” (211–215)
2. TTs’ lack of cohesive information management strategies made the development of strong team processes like communication, scientific coordination, and project management more difficult	Carla: • “So, when I first came on, I just was not totally sure, so I just kept a notebook of meeting minutes. Like I just wrote it down hard copy. I think the challenge with that is things never really got disseminated to the group, and then things got forgotten. And then you lose the notebook like a month in, and you’re at the next monthly meeting, and you’re like, “Shoot! What was that? Or whose job was that?” (166–170) Uma: • “So, it’s like how do I store all of these documents so that I can know which one is the most up to date? And also, I just don't quite know what to do with them, right? Like, I don't want my team members to be confused if they go in and say like, “Oh, which version is the most important, like the most recent version?”… But I guess that’s the biggest thing of, like, being able to find something or know where to store is how do you deal with multiple revisions and multiple versions and making sure you store them, but also that the team knows which one is right.” (349–351; 366–368). Ben: • “It’s sloppy, it’s messy… It’s really awful, and that’s been challenging. With this many people, we do have a Word document and, um, we’re often working off of 2 and consolidating 2 different manuscripts, which is, I just hate that so much. But the challenges with sending it from person to person is it’s gonna take forever.” (434–439)
3. TTs’ research was hindered by the institutional challenges of interdisciplinary team information sharing	Ben: • “This is, this is something that is probable unique, well, not appreciated by people who have, you know, PHI-encrypted servers. Instead, we have to jump through hoops to get this… It was a terrible process, and completely cumbersome.” (481; 489) • “I’m really often feeling like, unfortunately, sometimes on this island of nutritional sciences, trying to claw my way and swim my way to get into some of this clinical insight.” (33–34) • “I think the team science approach isn't truly functioning how it should be with that, with that project. It’s, it’s, yeah, unfortunate, right?” (45–46) Nancy: • “But, we, there are a few projects where we are collaborating with colleagues external to UW in the community at a, at a health system. And I know that it, uh, we were able to set it up so they could access the secure Box folder. But it was just really, it was, it was a lengthy process at the beginning. And, I mean, *I*, I understand why it’s necessary. We have to have security and everything, of course. But sometimes I just wish there was a more streamlined way of, of doing it, you know.” (260–265) Carla: • “Like if I’m trying to access something quickly from home because I’m thinking about it or I want to, like I’m writing something, it can be really painful. And also, like, the login and the password, and then if you like, you know, God forbid you use a different browser, and you don't save your password, and you’re like, “Oh, God, what was it?” I just think it’s the UW constraints, they’re not super…they’re a little bit cumbersome sometimes.” (158–162) Uma • “It’s just a lot of, like, admin, like…I don't know where I fall in this kind of research space. And also, my desk, my office is within the School of Pharmacy and within the SRC. So, kind of wearing multiple hats and trying to figure out resources from what’s…who’s the best person to contact for resources from all those different ones.” (329–332) Steve: • “You know, nobody tells you what tool you should use just to let you know. The supporting organization, in this case, ICTR, didn't tell me, “This is how you should store data.”” (126–127)
4. TTs benefitted from shared approaches to information management that foregrounded transparency, accountability, and trust	Katie: • “So, that’s when I realized we really need to make sure we’re filing and naming things, and there should be accountability basically. So, accountability is not just making sure this data is stored but making sure of who uploaded it and who verified it.” (242–244) Sarah: • “I often would not be able to find things if I didn't have a team that was very organized and knowing where they are. And I have a research program coordinator who’s been working on my team for 4 years, and she’s, she’s very fastidious and very talented.” (267–269)

### Theme 1: TTs’ Piecemeal, Reactive Approach to Information Management Created Conflict Within the Team and Slowed Scientific Progress

Participants described TTs that took a piecemeal, reactive approach to information management, which ultimately slowed scientific progress on their teams. TT members who encountered an information challenge would identify a solution, leaning heavily on their own personal approaches to information management and university-provided and supported platforms, without necessarily considering how the varying solutions integrated or conflicted with one another or how others on the team were approaching similar information management tasks. This lack of a unified approach to information management was “problematic,” in the words of one PI, because “each one of my staff members seems to have a preference for one thing over another” (Ben, 508–9). The consequences were significant, participants noted, because they often forgot where they stored or saved a crucial project detail without clear documentation. They said this caused confusion on their teams and wasted time.

Our participants explained that a key problem was that their research platforms and tools were numerous but not interoperable. A Research Scientist in our study described a utopian vision of information management, where “everything is reachable” from a single portal. But he worried how “there’s not really any platform successful to do that” precisely because the available tools did not speak coherently to each other (Alex, 243–5). Combined with the freelance approach to information management of every individual, this lack of interoperability imposed a number of administrative burdens on TTs.

These administrative burdens fell especially on the team’s leader, absorbing large portions of a leader’s time and consequently slowing a team’s productivity. This sentiment was best captured by PI Ben as the problems that “keep me up at night” (Ben, 515). Ben explained how he feels he’s “doing less and less science and more of just this type of administration, budgeting, that kind of thing.” To underline the point, Ben said, “the longer I’ve been here [at UW], the less and less science I’m actually doing” (Ben, 532–4). In short, PIs struggled to find time to conduct innovative science when their day-to-day tasks were consumed with the administrative responsibilities of being a *de facto* project manager without a cohesive approach to information management.

In these ways, TT members explained the reactive nature by which they developed processes for information management. For example, a Postdoctoral Researcher told us how her team did not at first consider how it was important that it saved files and documented decisions in a way that was transparent to everyone. “We didn't really explicitly care about transparency, but then we realized transparency really matters” (Katie, 212). Participants described how, without clear direction for a team when a project begins, the decisions of one individual influenced the information-sharing approach of the entire team. “Someone just started using PPT slides, so that’s when we started using PPT slides,” she noted. “And someone tried to share it through Box, and then we started using Box as a platform to share it.” She added, “I don't think we really talked about how we’re going to share it within the team. It just naturally happened” (Katie, 136–7). This passive approach to letting information sharing “naturally happen” on teams often caused confusion and delays for busy TT members.

### Theme 2: TTs’ Lack of Cohesive Information Management Strategies Made the Development of Strong Team Processes Like Communication, Scientific Coordination, and Project Management More Difficult

Team processes such as communication, scientific coordination, and project management all rely on the ability to share information across platforms, departments, and projects. The piecemeal information management approaches TTs developed, as described in Theme 1, further eroded their ability to develop strong team processes that supported and facilitated their scientific work.

Participants noted a lack of systematized sharing of information across the TT and highlighted how it hindered team communications. Carla, a PI, shared that when she started doing research at UW, she would write all of her notes in a physical notebook. But she soon realized the “challenge with that is things never really got disseminated to the group, and then things got forgotten” (Carla, 167–8). Further, a few months into the project, she lost the notebook, which included valuable project data that had not been communicated to the rest of the team. She said the incident spurred her to develop a more systemized approach to information management on her team, and now they have a clear policy. But she expressed frustration that she did not push her team to address these issues at the project’s outset, which would have prevented the loss of vital data.

Even scientific tasks that were relatively straightforward for an individual, such as naming and saving files or writing a manuscript, became complex once being done across a team. A Postdoctoral Researcher described the conundrum on her team over how to clearly save different versions of a file: “So, it’s like how do I store all of these documents so that I can know which one is the most up to date?” Because no one on her team had established a clear process, she didn't “quite know what to do with them.” Her concern, she continued, was with the potential confusion this would cause her teams. “I don't want my team members to be confused if they go in and say, “Oh, which is the most recent version?”” The issue, she said, was dealing with “multiple revisions and multiple versions” of documents and “making sure you store them” in a manner that “the team knows which one is right” (Uma, 349–51, 367–8). Similarly, when asked about consolidating manuscripts from multiple versions in Word, a PI loudly sighed and then chuckled, calling it a “sloppy” and “messy” process (Ben, 434).

### Theme 3: TTs’ Research Was Hindered by the Institutional Challenges of Interdisciplinary Team Information Sharing

TT members described the institutional structures of the research university, in which different schools and colleges, or even departments within a school, may have vastly different tools available to researchers or different approaches to information security. These differences created conflict among interdisciplinary researchers working across the university, hindering the smooth conduct of translational research. Several participants described how these differences created a tension in information management between safeguarding the security of data, especially protected health information (PHI), and easily sharing team information that may not need the same level of security. Participants expressed frustration with the challenge of storing and sharing files in a manner that preserved PHI without creating too many administrative hurdles for teams to overcome. Further, while the UW’s Informational Technology website offers a number of general tools related to collaboration and coordination, these resources are not designed with the problems of clinical and translational researchers in mind and no participants in our study mentioned them or seemed aware of their existence. These challenges underscore the institutional factors beyond a team’s control that can negatively impact its approach to information management.

Multiple participants described being ensnared in a bureaucratic web that made it difficult to set up information management systems. For example, a PI said he had to “jump through hoops” to get a secured server space, which he called “a terrible process, and completely cumbersome” (Ben, 481, 489). A Research Scientist expressed a similar sentiment when she talked about the “lengthy process” of setting up a secure Box folder. “Sometimes I just wish there was a more streamlined way of doing it,” she said (Nancy, 253, 263). Another PI described how “really painful” it can be to deal with security restrictions when trying to quickly access a key piece of information while in the midst of an important task such as data collection or analysis. “God forbid you use a different browser, and you don't save your password,” she said (Carla, 159–161).

Further, disciplinary silos can also impact information sharing. A Basic Scientist PI explained the divide on his team between the clinical and basic science camps that impeded the free flow of information. Sharing PI responsibilities with a clinical partner, he said he had less control than he would like over the team’s overall culture of information management due to his multi-PI status. He said he often felt stuck on the “island of nutritional sciences, trying to claw my way and swim my way to get into some of this clinical insight.” Because of this, “the team science approach isn't truly functioning how it should be with that project,” which he called “unfortunate” (Ben, 33–4, 45–6).

Other participants discussed the challenges of figuring out how to share information easily in an environment that did not provide clear and accessible guidance for researchers about how to carry out their translational projects. While the UW’s IT department provides general resources to help teams collaborate, it does not provide any specific guidance or tools that address the particular challenges of clinical and translational research. “I don't know where I fall in this kind of research space,” a Postdoctoral Researcher told us. She said she struggles with “wearing multiple hats and trying to figure out who’s the best person to contact for resources” from the many departments associated with her position and grant (Uma, 330–2). In the words of one PI, “nobody tells you what tools you should use” (Steve, 126). Steve’s words reflect how he was never given any university guidance for information management on science teams. Without such guidance, participants said they spent considerable time coming up with solutions on their own, including figuring out new systems, how to request access, and how to train new members.

### Theme 4: TTs Benefitted From Shared Approaches to Information Management That Foregrounded Transparency, Accountability, and Trust

The few participants whose TTs did have cohesive team-wide strategies for information management described a resultant culture of transparency, accountability, and trust, team characteristics that have been shown to improve scientific outcomes [6]. One such strategy included codifying and distributing meeting agendas and related task assignments for all members. A Postdoctoral Researcher told us how she used one document that combined an archive of meeting agendas/notes and task assignment information, which she updated in real time for her team. This document allowed her to present information to the team, keep track of who was responsible for what, make notes on next steps, and follow-up with specific team members, as appropriate. At the same time, all team members had access to the file, which promoted transparency and accountability on her team.

A theme that many participants brought up as a countervailing force to information loss was accountability or the assurance you can rely on your team members to accomplish their tasks and to follow team procedures. After a Postdoctoral Researcher we interviewed discussed a missing element during data collection, she told us, she “realized we really need[ed] to make sure we’re filing and naming things, and that there should be accountability basically.” To her, that meant expanding the definition of accountability to include “who uploaded [information to a server] and who verified it” (Katie, 242–4).

One PI, Sarah, framed her ability to find things easily as a product of the trust she had in her efficient and collaborative team, notably their clearly defined and shared approach to project management. She gave particular credit to a long-time research coordinator, whom she described as “very fastidious and very talented,” who had been in the same position for over four years (Sarah, 269). Moreover, she said, her team had created a lively and collaborative group chat in Microsoft Teams, where busy members could pool team knowledge to find quick solutions to outstanding questions.

As Sarah explained, a culture of trust was built through the responsible and thoughtful information-sharing practices of TTs. Sarah stressed the importance of intention and clarity when sharing information with team members. She said that information should be shared with a clear purpose, and team members should consider if they were sharing the right information with the right person at the right time. Clarity meant stating what you wanted team members to do with the information you were sharing or presenting. Even with an email, this included identifying people by name, listing specific tasks, and setting clear expectations. These practices, she noted, worked in the aggregate over the long term to improve her team’s culture of trust by showing members how you valued their time and contributions.

But clear practices rooted in transparency, accountability, and trust, we discovered in our study, were the exception rather than the rule. Many participants, especially those on larger teams, did not share information as freely or coherently as this, even though doing so is a relatively low-cost way to make a big impact on a team’s functioning.

## Discussion

Overwhelmed and overloaded with responsibilities, TTs encounter a multitude of information management challenges. Faced with university-provided default tools generally not designed for supporting scientific work, TT members first try to scale their personal information management strategies or simply adopt what someone else on their team is doing. Each new information challenge is met with an independent solution, one that rarely accounts for other existing team tools or processes, further complicating and hindering the team’s scientific work. There is no one-size-fits-all solution, but the current environment in which TTs are building *de facto* information management solutions without guidance or support from their institutions results in wasted time and effort, frustration, and lower scientific productivity. Yet TTs that do create more thoughtful, proactive approaches see increased transparency, trust, and accountability, characteristics associated with successful science teams [6], providing some clues for how TTs can implement better approaches to information management in such contexts of high-task interdependence.

While we found that some TTs devised innovative approaches to information management, most waste time and resources building information solutions in a reactive manner. Overwhelmed with the sheer volume of both scientific and administrative tasks required in scientific work, TTs, and especially PIs, do not invest time in proactive and codified approaches to their team’s information management. As our work has shown, this is, in part, because most don't see the centrality of information or their information behaviors to the team’s overall research process [19]. As a result, they act as *freelance information management agents*, whose individual choices conflict and sow confusion on the team level, ultimately hindering team productivity and slowing the translation of science.

While this type of high-task interdependence has typically been examined in the SciTS field from the vantage point of integrating *scientific* tasks, our study reveals how scientific tasks have quotidian *informational* dimensions that must also be integrated, such as how a team handles version control on a document when you have multiple collaborators co-creating. In these ways, our study shows how the lack of proactive discussions about how to manage a team’s information causes confusion and delays that have ramifications on a team’s efforts to establish sound team processes. As such, the results of our study suggest a reciprocal relationship between information management and strong team processes, providing some preliminary evidence that when TTs develop shared protocols for information management, they benefit in terms of transparency, trust, and accountability. How, then, can the fields of SciTS and Translational Science help?

**Figure 3.**
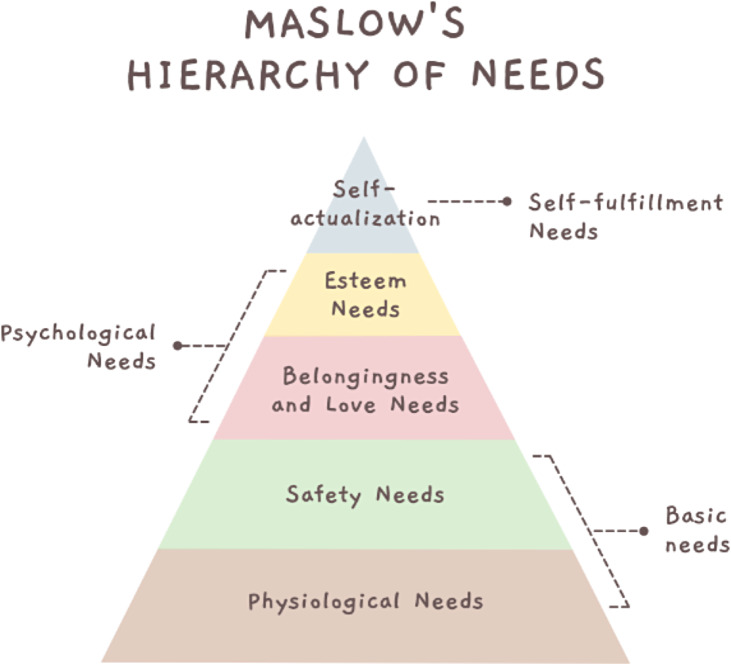
Maslow’s Hierarchy of Needs [23].

As one of the most “cognitively contagious ideas” in the behavioral sciences, Maslow’s hierarchy of needs (Fig. 3) provides a helpful model for reconceptualizing our existing SciTS interventions [22]. Maslow’s hierarchy is typically seen as operating on a principle of *cognitive priority*: when a poet is starving, the beauty of her sonnets is of little interest because hunger takes cognitive precedence over self-actualization. But the hierarchy also signals *developmental priority*: when a baby is starving, self-actualization is of little interest because she has not yet developed the desire to seek its fulfillment. The connection for TTs is clear: information management is essential because it frees up cognitive space for TT members. That freed up cognitive space, in turn, allows for teams to mature and develop the kind of collaborative spirit they need to produce groundbreaking science. At the same time, the hierarchy suggests that interventions must be designed for the appropriate developmental level of a team: a team that lacks a sound approach to information management will be less capable of building a psychologically safe environment.

**Figure 4.**
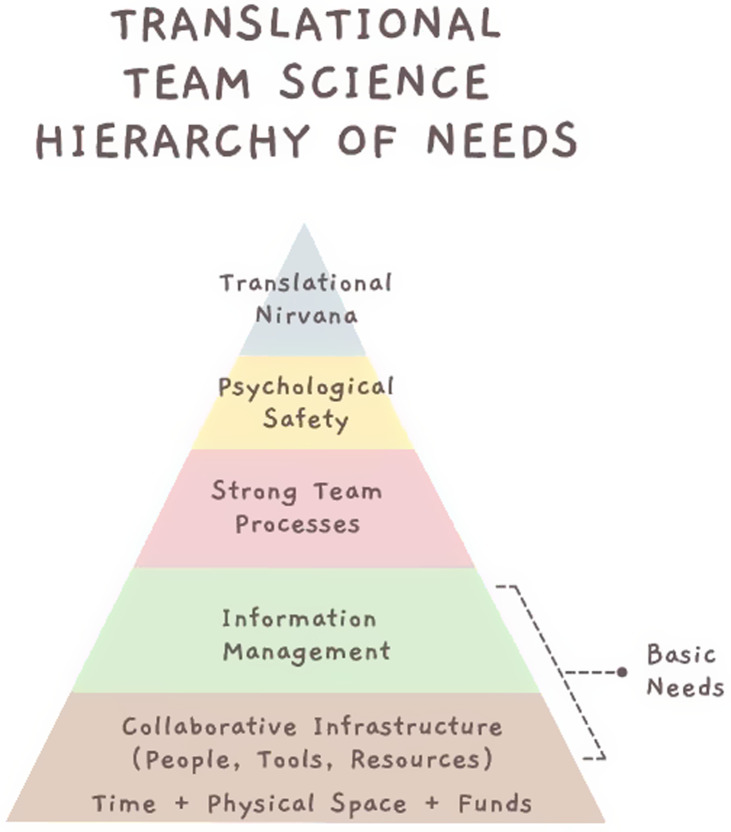
Translational Team Science Hierarchy of Needs.

Following Maslow, we hypothesize that team processes need to be established in a sequence that respects what we call a *Translational Team Science Hierarchy of Needs* (Fig. 4). Like Maslow’s hierarchy of needs, where basic physiological conditions must be met before one reaches the apex of self-actualization, translational teams – made up of discrete individuals with unique approaches to managing their information – first need a harmonized approach to information management before they can achieve the full promise of their scientific vision.

[22] For these reasons, we placed information management near the base of the *Translational Team Science Hierarchy of Needs*. As one of the “basic needs” that TTs need to operate, information management sits at a critical juncture in the hierarchy, analogous to the “safety needs” of humans as described originally by Maslow. Beyond the basic infrastructure that TTs need to operate, we see information management as the lifeblood of highly effective TTs because it is foundational to the development of strong team processes. As we suggest here, TTs will not be able to progress up the hierarchy toward the apex – what we label here a culture of psychological safety that enables a “translational nirvana” – without first establishing sound information management processes. In short, TTs will not be able to collaborate effectively if they can't share information coherently.

How, then, might the SciTS and Translational Science fields begin to address these information challenges? One approach would be to consider how existing Team Science interventions incorporate elements that foster sound information management practices. Here at UW-Madison, for example, our Collaboration Planning sessions with TTs explicitly ask teams to proactively decide how the results of meetings and communications will be documented so that they will be accessible to everyone on the team, including any external collaborators such as community partners. We also encourage TTs to think about where the array of types of information (e.g., meeting notes, SOPs, forms, tasks, contact lists) will live and how they plan to document and train everyone on the team on these processes. Our evaluation results from these sessions show that TT members appreciate discussing these often-neglected questions about information management because they quickly understand how they will benefit the team in the long run [15]. Given our participants’ lack of knowledge of available tools and guidance for implementing them in collaborative environments, our study suggests another effective intervention would be for universities to create resources that onboard and orient teams to using such tools collaboratively.

But beyond simply modifying what we currently offer, we believe our study provides a new way to think about Team Science interventions with an eye on what is most crucially needed by TTs in the present. We suggest that the SciTS field has, at times, reached for complicated solutions and technological fixes rather than considering the baseline of what TTs need. Given the accumulated time and energy wasted searching for files and discarded information, as well as the impact of that lost time on scientific progress, we imagine a new frontier of interventions that are designed with an emerging *Translational Team Science Hierarchy of Needs* in mind. In this model, interventions will be “right-sized:” designed to maximize our return on, while minimizing the burden of, our limited time investments.

### Limitations

The current study has two key limitations. First, the number of participants interviewed was small and represented just four categories of TT members. Second, the study took place at one large Midwestern research institution with an active CTSA and Team Science program; as such, the results may not generalize to TT members in other environments. However, as the first study of the information behaviors of TTs of which we are aware, we believe these results create a starting place for learning more about this topic and advancing dialogue and future research.

### Future Directions

Our study seeks to begin an important conversation about how best to promote information management on TTs. We envision a number of fruitful avenues for additional research. One approach will be to deepen our study by interviewing more of the *personas* identified by Gonzales et al. (2020). This additional work will allow us to better understand the information behaviors of various TT roles and whether, and to what extent, they differ from some of the most standard roles we explored here. A second approach will be to identify existing evidence-based SciTS interventions beyond UW-ICTR’s Collaboration Planning that improve a team’s approach to information management. As we suggest here, any proactive team discussion is helpful given the current state of affairs where almost none takes place, and the more transparently and clearly a team documents its plan, the better. A third approach might consider the role of our institutions and organizations in providing solutions and strategies for TTs. What types of toolkits, processes, and prepackaged solutions might institutions readily provide to TTs? How might they streamline some of the bureaucratic and administrative processes in order to save TTs money and time in the long run?

## Conclusion

To our knowledge, this is the first study to analyze the challenges that information management imposes on the conduct of team-based Clinical and Translational Research. We identified how TTs’ piecemeal and reactive approaches to information management impeded their efforts to build strong team processes, approaches that, without guidance or support from the siloed university, ultimately slowed scientific progress on their teams. We also identified some promising solutions for teams, which include shared and codified approaches to information management that emphasize transparency, trust, and accountability. We encourage the SciTS and Translational Science fields to address the challenges of information management on TTs through additional research to understand the information behaviors of TTs. Finally, we propose a new model for the SciTS field – a *Translational Team Science Hierarchy of Needs* – that suggests new considerations for the design, development, and evaluation of interventions, with a focus on targeting the appropriate stage of team development. Such a focus has the potential to help TTs create a strong base that supports team processes that maximize a team’s scientific potential.

## Supporting information

Kelly et al. supplementary material Kelly et al. supplementary material

## Supplementary material

The supplementary material for this article can be found at https://doi.org/10.1017/cts.2023.614.
