# Supplementary material for: Toward a translational team science hierarchy of needs: Exploring the information management challenges of team science
Source: J Clin Transl Sci. 2023 Aug 23;7(1):e210. doi: 10.1017/cts.2023.614 (PMC10603362; doi:10.1017/cts.2023.614)
Supplement: Kelly et al. supplementary material [file S2059866123006143sup001.docx]

**Supplementary Material**

*Appendix A (recruitment email)*

Hello _________,

The UW Institute for Clinical and Translational Research is seeking members of translational research teams who are at least 18 years old to participate in a research study. Your information was identified through a search of research teams at the University of Wisconsin-Madison. The purpose of this study is to better understand how the information strategies and processes of translational teams impact their ability to achieve their scientific objectives.

Participation in this study is voluntary and involves completing a 1-hour interview that will take place at the UW Institute for Clinical and Translational Research.

For more information or to participate in this study, please contact the principal investigator, Dr. Betsy Rolland, via phone at 608-265-3185 or via email at [brolland@wisc.edu](mailto:brolland@wisc.edu)

Thank you,

(Signature)

Study Title: Understanding Information Strategies and Processes of Translational Teams

*Appendix B (interview questions)*

1. Can you briefly explain your project? What is your team trying to achieve?
2. What individuals, groups, or organizations are included in the project?
3. What is your role on this project?
4. What are the activities involved in your role?
   1. From start to finish, what did you do to complete this activity? (Ask for 2-3 activities)
      1. Who did you need to collaborate with?
      2. What tools did you use?
   2. What were the outputs of the activity? (Ask for 2-3 activities)
      1. Who else used the outputs?
      2. Where did you keep the outputs?
      3. How were the outputs shared?
      4. How did you decide how and with who to share this output?
5. Tell me about a time when you couldn’t find something you needed
6. Tell me about a time when you didn’t know where to store or how to share something
7. What are the expected overall outputs for the project? (Publications, datasets, presentations, etc.)
8. Is there anything else you want to tell me about your work?
